# The Foreign Journals: Surgery

**Published:** 1838-10

**Authors:** 


### SURGERY.

### *On Chopart's Method of Amputating the Foot.* By M. Blandin.

At the sitting of the Academy on the 29th of May, M. Blandin presented a memoir on the amputation of the foot at the articulation of the os calcis and astragalus with the scaphoid and cuboid bones. This operation, so desirable in cases of disease of the tarsal bone, has been generally objected to on account of the danger of retraction of the calcaneum; the action of the tendo-achillis being no longer counterbalanced by the tibialis and peroneus anticus, the tendons of which are divided in the operation. M. Blandin has performed the operation several times with success, and his expectations have never been disappointed by the retraction of the heel, and consequent depression of the cicatrix. Having recently had the opportunity of examining the stump in three individuals who have undergone this operation, he thus describes the anatomical disposition of the parts: "The tendons of the anterior muscles of the leg present, at the cut extremity, a very curious disposition, which has not hitherto been noticed. These tendons terminate upon the head of the astragalus and upon the cuboidal surface of the calcaneum, which have been deprived of their articulating cartilages by absorption; they there contract a very important insertion, which enables them to continue to transmit to the foot the action of the muscles to which they belong. In addition to this, by means of the cicatrix, they are continuous with those of the superficial muscles of the sole, which act upon the calcaneum by their remaining insertion; so that the anterior muscles of the leg, and those of the sole of the foot, form but a single system, the different parts of which concur in preventing the elevation of the heel, and enable the foot to have the degree of flexion necessary for the functions which it is still called upon to perform. So that we have, in fact, *digastric* muscles, the middle tendons of which are formed by those of the dorsal and plantar muscles united, whilst the opposed bellies are constituted by the fleshy fibres of the anterior muscles of the leg and the inferior of the foot. A bursa mucosa, formed of the remains of the synovial membranes of the articulation destroyed, is placed beneath the middle tendons of these anormal digastrics, and facilitates their motion upon the astragalus and calcaneum. We find these two powers opposing the retraction of the heel, which would otherwise appear inevitable: 1st, the new insertion of the anterior muscles of the leg into the calcaneum and astragalus; 2d, the continuity accidentally established by means of the cicatrix between the anterior muscles of the leg and those of the sole, giving the former an additional adhesion upon the inferior surface of the heel, and thus enabling them to counterbalance the action of the muscles of the calf." M. Blandin concludes by stating that retraction of the heel after this operation is rare, and would probably recur still more rarely if care were taken to preserve the tendons sufficiently long: he gives his decided testimony in favour of the operation.

In the discussion which followed, M. Larrey denied the general utility of Clopart's operation, and stated that he had seen the retraction of the heel so great that the patient could only walk by supporting the knee on an artificial leg. M. Velpeau considered the retraction as of rare occurrence, but had seen two cases in which it had produced much inconvenience. M. Blandin, in reply, stated that he had performed the operation eleven times without the occurrence of this accident. *Bulletin de l'Academie. June,* 1838.

---

*On Rupture of the Perineum in Females.'* By Professor DIEFFENBACH, of Berlin.

IN this important paper Professor Dieffenbach first deprecates the general practice of leaving slight ruptures of the perineum to the unaided efforts of nature. On examining the perineum after the healing of a rupture extending backwards for half an inch or a whole inch, no corresponding cicatrix is seen, but the perineum is found contracted and diminished in breadth, and the labia are drawn backwards in such a manner as to enlarge the orifice of the vagina. In slight cases this is of little consequence; but, when the rupture has been extensive, relaxation of the vagina probably ensues, with protrusion of its mucous coat, and frequently also prolapsus of the uterus. In straining at stool, the anterior wall of the rectum bulges forwards into the vagina; and in other instances the superior wall of the vagina and neck of the bladder sink into its cavity. The fæces are still retained, but flatus often escapes unexpectedly. But still these evils are small compared with those which ensue in cases where the perineum, vagina, and rectum have been torn in such a manner that the vagina and rectum form but one cavity. Professor Dieffenbach recommends, therefore, that, even in slight cases of rupture, the edges of the wound should be stitched together, and union sought by the first intention, in order to avoid deformity of the parts. The following cases are given in illustration.

CASE I. A young woman, aged twenty-six, in giving birth to her first child, suffered from rupture of the perineum, extending backwards about an inch. The edges of the wound were cleaned, and three sutures applied. Two of the sutures were removed on the third day, and the other on the fourth. The union was complete.

CASE II. The perineum of a woman, aged thirty, was torn to the extent of an inch and a half, in giving birth to her third child. Ten hours after the accident the sutures were applied, and were removed on the fourth and fifth days; complete reunion having been effected.

[Two similar cases are detailed, which we will not transcribe.]

CASE V. In a young woman, aged thirty, who had led a very irregular life, there was found incipient prolapsus of the uterus; the genital organs were very much relaxed and very large, and the perineum was extremely narrow. It could not be ascertained whether this state was the consequence of simple dilatation, or whether rupture of the perineum had previously existed. The posterior edges of the labia were made raw, and then approximated and retained in situ by means of eight sutures. Two additional sutures were employed to unite the mucous membrane of the vagina. The operation was completely successful: the perineum acquired considerable breadth, and the vagina became so narrow that, after her recovery, the patient resumed with great success her previous vocation.

CASE VI. In a strong, robust young woman, aged twenty-six, in labour with her first child, the perineum, part of the vagina, and several inches of the rectum, were unfortunately torn. Six or eight hours after the accident, a suture was passed through the edges of the vaginal wound, and by means of it the edges of the wound in the posterior wall of the vagina were brought into view and united. In the same way the wound in the rectum was drawn forwards and stitched, and the parts were then allowed to resume their natural position. The perineum was then united by powerful sutures, and moderate antiphlogistic treatment pursued. The obesity of the patient was a great obstacle to the success of the operation: it succeeded so far, however, that the cavities of the vagina and rectum were separated from each other, and the power of retaining the fæces restored; but the reunion of the greater part of the perineum did not take place.

[Four other cases are given: two of them were of recent occurrence, the other two had existed for years. In the former two, the operation was completely successful; and partial success was obtained in the latter.]

The treatment pursued was in general antiphlogistic, and, during the first six, eight, or ten days after the operation, the bowels were kept constipated by repeated doses of opium. When passage of the bowels could no longer be avoided, an elastic catheter was introduced into the rectum, and a stream of warm water, holding a small quantity of soap in solution, was thrown up; by which means the scybalous mass was broken, and passed without injuring the parts. The catheter was employed, in general, four times daily to empty the bladder.

*Medicinische Zeitung.* No. 52. 1837.

---

### *On the beneficial Action of Acetate of Lead in incarcerated Hernia.*
### By Dr. HUXTHAUSEN.

CASE I. A woman, aged seventy-six, weakened by chronic cough, and having an unreduced inguinal hernia, was suddenly attacked with severe cutting pains in the bowels, accompanied by vomiting, at eleven o'clock in the evening of the 7th May, 1837. She was seen by her medical attendant at nine o'clock on the following morning, who found the symptoms to be dependent upon the hernia having become incarcerated. It was about the size of a hen's egg, hard, and painful upon pressure. As the attempts at reposition failed, an injection of eight ounces of a solution of acetate of lead (Acet. plumb. ℈j., Aq. ℥vj.) was administered, a spoonful of castor oil given internally, and ice applied to the tumour. At twelve o'clock the reposition followed of its own accord.

CASE II. A robust woman, aged thirty-two, affected with chronic crural hernia, was very subject to hysterical attacks. On the 13th and 14th September, 1837, she had two violent paroxysms, in consequence of which the hernia became incarcerated. The tumour was of the size of a goose's egg, distended and painful. All attempts at reposition having failed, eight ounces of the solution of the acetate were given in an injection, ice was applied to the tumour, and a powder, composed of acetate of morphia, gr. $\frac{1}{8}$; nitrate of bismuth, gr. iii.; sugar, ℈ss.; and chamomile oil, gtt i., administered every hour. The ice was removed in half an hour, as it seemed to increase the pain. From this time the symptoms were relieved, the vomiting ceased, the tumour gradually decreased in size, and in five hours the reposition was complete.

CASE III. A boy, aged twelve, fell on the evening of the 15th October; and, when seen on the following morning, complained of great pain in the right inguinal region, and was in a state of high fever. On examination, an incarcerated hernia, about the size of a goose's egg, hard and extremely painful, was detected. Reposition was attempted, but failed: twelve leeches were then applied to the tumour, a spoonful of castor oil given internally, and six ounces of the solution of the acetate administered in injection. At eleven o'clock, little benefit resulting from these measures, six ounces additional of the solution of the acetate were given in injection. Improvement now took place so rapidly that at one o'clock spontaneous reposition ensued.

[Although the small number of cases here given, and the impossibility of exactly measuring the influence of the other agents employed, forbid us to draw positive conclusions as to the efficacy of this mode of administering lead in hernia, still the subject deserves the attention of surgeons.]

*Medicinische Zeitung.* No. 7. 1838.

---

*On Hemlock Baths in Cutaneous Diseases.* By Dr. Fantonetti.

M. Fantonetti has proved the good effects of hemlock baths in acute and chronic affections of the skin: he has used them with success in erythema, impetigo, psoriasis, lichen, and they have even alleviated the pains of gout. He regards the baths and lotions of the decoction or infusion as sedative, resolutive, and desiccative. This remedy acts promptly, and, used in a proper manner, never produces injurious effects. The bath is prepared either by infusing or boiling eight or ten small handfuls (*pincées*) of dried or fresh hemlock in eight or ten quarts of water, which is to be then poured into the bath, heated to the temperature of 90° or 92° Fahr. The patient should remain in the bath an hour or two, and should be surrounded by a blanket brought close around the neck, to prevent the vapour producing vertigo or pains in the head. M. F. supposes the hemlock to act from the alkaloid principle which it contains, and which explains, he asserts, the reason why the decoction and the infusion are equally efficacious. This alkaloid principle does not evaporate in the same manner as the volatile parts of aromatic plants, which are employed for similar purposes.

*Revue Médicale. October*, 1837.

---

*Dislocation of all the Metatarsal Bones upon the Tarsus.* By M. Mazet.

The wheel of a heavy laden cart passed over the foot of a young man, aged nineteen. When examined, the foot appeared twisted on itself, the convexity of the curve being above and outwards. On the dorsal surface of the foot was a wound, across which was a bony projection, elevating the extensor brevis digitorum, and the tendons of the common extensors of the toes, apparently formed by the posterior extremities of the three middle metatarsal bones. External to this projection was another, but this was covered by skin. The bones of the leg were not broken, and the toes were uninjured. It being believed that several bones were bruised, the leg was amputated. The patient died of phlebitis. The amputated limb was dissected, and the following were its appearances: The most external digitation of the extensor brevis was torn; the tendon of the tibialis anticus was partially ruptured, about an inch from its termination; none of the muscles in the sole of the foot were at all torn; but the interossei, contained in the first and in the fourth space, were ruptured. All the metatarsal bones were dislocated from their tarsal articulations, although they were not displaced together towards the same point, and did not maintain their normal relations to one another: the second, third, and fourth were dislocated together towards the dorsal surface, so that their tarsal extremities rested on the superior surfaces of the cuneiform bones; the first and the fifth were quite separated from their usual connexion, and were situated thus: the posterior extremity of the first rested on the inner side of the first cuneiform bone,—that is to say, it was dislocated inwards, carrying with it and stretching the tendon of the peroneus longus muscle; the fifth metatarsal bone, which had suffered the most considerably, was dislocated at its posterior extremity, which is not connected to the bones of the tarsus by either tendon or ligament. This bone was turned on its axis, so that its internal surface was upwards; and, in addition, it was fractured about three-quarters of its length from its tarsal articulation.

*Gazette des Hôpitaux. Février*, 1838.

